# In Vivo and In Vitro Evaluation of the Feasibility and Safety Profiles of Intraarticular Transplantation of Mitochondria for Future Use as a Therapy for Osteoarthritis

**DOI:** 10.3390/cells14030151

**Published:** 2025-01-21

**Authors:** Carlos Vaamonde-Garcia, Tamara Hermida-Gómez, Sara Paniagua-Barro, Elena F. Burguera, Francisco J. Blanco, Mercedes Fernández-Moreno

**Affiliations:** 1 Grupo de Investigación en Reumatología (GIR), Instituto de Investigación Biomédica de A Coruña (INIBIC), Complexo Hospitalario Universitario de A Coruña (CHUAC), 15005 Sergas, Spain; carlos.vaamonde.garcia@sergas.es (C.V.-G.); tamara.hermida.gomez@sergas.es (T.H.-G.); sara.paniagua.barro@sergas.es (S.P.-B.); elena.fernandez.burguera@sergas.es (E.F.B.); 2 Grupo de Investigación en Reumatología (GIR), Instituto de Investigación Biomédica de A Coruña (INIBIC), Complexo Hospitalario Universitario de A Coruña (CHUAC), Universidade de A Coruña (UDC), 15006 A Coruña, Spain; 3 Grupo de Investigación en Reumatología y Salud (GIR-S), Departamento de Fisioterapia, Medicina y Ciencias Biomédicas, Facultad de Fisioterapia, Centro Interdisciplinar de Química y Biología (CICA), INIBIC-Sergas, Universidade de A Coruña (UDC), Campus de Oza, 15006 A Coruña, Spain; 4 Centro de Investigación Biomédica en Red, Bioingenieria, Biomateriales y Nanomedicina (CIBER-BBN), 28029 Madrid, Spain

**Keywords:** mitochondria isolation, mitochondria injection, chondrocyte, cartilage, synovium, osteoarthritis, in vitro model, in vivo model

## Abstract

Osteoarthritis (OA) is the most common rheumatologic disease and a major cause of pain and disability in older adults. No efficient treatment is currently available. Mitochondrial dysfunction in chondrocytes drives molecular dysregulation in OA pathogenesis. Recently, mitochondrial transfer to chondrocytes had been described, enabling transplant of mitochondria as a new avenue to modify the OA process, although evidence on its feasibility and safety remains limited.The primary objective of this study was to demonstrate the feasibility and safety of intra-articular mitochondrial transplantation. Mitochondria were isolated from liver using the procedure described by Preble and coworkers combined with magnetic beads coupled to anti-TOM22 antibodies. The organelles obtained were analyzed to determine their purity and viability. The safety and viability of the administration of the isolated mitochondria into articular tissues as well as the integration and distribution of the transplanted mitochondria within joint tissues were analyzed using both in vitro and in vivo models. We established an efficient, reproducible, effective, and rapid protocol for isolating mitochondria from liver. We obtained mitochondria with high viability, yield, and purity. The isolated mitochondria were injected into joint tissue using both in vitro and in vivo models. Functional mitochondria were detected in the extracellular matrix of the cartilage, menisci and synovium. Our results establish a safe and viable protocol for mitochondrial isolation and intra-articular injection. The methodology and findings presented here pave the way for future studies in osteoarthritis models to validate mitochondrial transplantation as a potentially effective treatment for OA.

## 1. Introduction

Osteoarthritis (OA) is a leading cause of impaired joint function and disability, associated with significant treatment costs and socio-economic burdens. Although current treatments for OA can improve symptoms, they do not inhibit the progression of the disease. In recent years, several research groups have focused their efforts on developing new treatments for OA, aiming to minimize pain, inflammation, and the degradation of articular tissues. Both in vitro and animal model studies of OA have shown promising results. However, few newly developed agents have successfully completed clinical trials [1], and OA treatment remains challenging, with no effective drugs currently available.

Mitochondrial dysfunction plays a crucial role in the pathogenesis of OA [2,3]. The term “transfer” refers to a process in which organelles are donated from one cell to another via various mechanisms [4,5,6]. Mitochondrial transfer may occur in response to a “help me” signal from a damaged cell [7,8], involving the formation of tunneling nanotubes or extracellular vesicles [9]. Intercellular organelle transfer has been described in both in vitro and in vivo model studies [10,11,12], suggesting that cell damage is critical in mitochondrial transfer [13]. The discovery of mitochondrial transfer has advanced treatments for mitochondrial dysfunction-related diseases and indicated its potential to restore function in damaged cells [8,14,15]. Mitochondrial transplantation involves augmenting or replacing damaged native mitochondria with viable mitochondria isolated from non-damaged tissue [16]. Studies in cell culture, isolated perfused organs, in vivo large animal models, and human clinical studies, suggested that mitochondrial transplantation is effective and safe [17,18,19,20].

Mitochondria play a pivotal role in cellular calcium (Ca^2+^) metabolism, and Ca^2+^ contributes to OA pathogenesis, inducing inflammation and cartilage degradation [21]. A recent study showed that intramuscular injections of mitochondria into the hind limbs of mdx mice reduced calcium deposits in muscles [22]. It could be intriguing to evaluate the potential impact of mitochondrial therapies in this context. Mitochondrial transplantation, already investigated in ischemic cardiac muscle [23], raises questions about how donor mitochondria survive high extracellular Ca^2+^ concentrations, given that skeletal muscle mitochondria are damaged by Ca^2+^ overload [24,25]. However, studies suggest that a paracrine response and increased membrane permeability induced by Ca^2+^ shock may be central to the therapy’s efficacy [24].

Emerging evidence indicated that an imbalance in mitochondrial homeostasis contributes to the incidence and progression of OA [3,26,27,28]. A recent study suggested that bone marrow–derived mesenchymal stromal cells (BM-MSC) can be used to transfer mitochondria to murine OA chondrocytes, thereby enhancing ATP production [29]. Other studies have demonstrated that therapeutic interventions targeting mitochondria alleviate cartilage damage and OA symptoms [30], suggesting that the transfer of healthy mitochondria is a potentially beneficial strategy for treating OA [29,31]. Lee and coworkers examined the therapeutic potential of mitochondrial transplantation for treating OA in a rat model and reported that mitochondrial transplantation ameliorated OA progression and reduced pro-inflammatory changes [32]. Recently UC-MSC-derived mitochondrial transfer restored metabolic function, mitochondrial dynamics, and resistance to oxidative stress in OA chondrocytes, improving some disease outcomes in an OA mouse model, highlighting mitochondria’s potential as a novel OA treatment [33].

However, none of these studies have investigated the fate of the transplanted mitochondria, whether they exit the joint and travel through the bloodstream to the liver or spleen, which specific tissues within the joint they integrate into, and if they do integrate, whether the mitochondria remain viable and functional, as well as the duration of their viability within the tissues. Given the limited evidence on the efficacy of mitochondrial transplantation for OA, this study aimed to evaluate the feasibility, safety, and potential of intra-articular mitochondrial transplantation in OA.

A critical aspect of mitochondrial transplantation for disease treatment is the method of isolation. Standardizing a rapid, simple technique that ensures a high yield of viable mitochondria will be essential. Various methods, often involving homogenization and/or differential centrifugation (DC), have been described, with earlier techniques forming the foundation of current practices [34,35].

Although DC-based methods are simple, they have several limitations, such as low purity of mitochondrial fractions [36]. Furthermore, DC protocols involve manual homogenization and repetitive steps that increase the time of isolation and the likelihood of mitochondrial damage, ultimately reducing mitochondrial viability [37]. The use of magnetic beads coupled to anti-TOM22 antibodies enables the isolation of highly pure functional mitochondria with minimal non-mitochondrial contamination [37,38,39]. The combined use of homogenization and DC to isolate mitochondria for transplantation into myocardial tissue was described [40,41]. This protocol required less time and allows for the uniform homogenization of the tissue, resulting in the isolation of highly purified and viable mitochondria.

This study aimed to develop and standardize a protocol for isolating high quality, viable mitochondria specifically for application in articular tissues. Additionally, it will evaluate the feasibility, safety, and potential of intraarticular mitochondrial injection in joints, studying the integration, distribution, and effects of transplanted mitochondria within joint tissues using both in vitro and in vivo models.

## 2. Materials and Methods

### 2.1. Animals

Animals were bred at the Experimental Surgery Unit of A Coruña under standard conditions. Animal experimentation was carried out in accordance with legislation regarding the protection of animals (European Directive 2010/63) used for scientific purposes and in compliance with the ARRIVE guidelines.

### 2.2. Mice

Twelve-week-old male C57BL/6JOlaHsd and NZB/OlaHsd mice (*Mus musculus*) were obtained from ENVIGO RMS. The age of the mice corresponded to that used in the literature to study knee alterations, including OA [42,43]. The mice were housed at the Experimental Surgery Unit of A Coruña under standard conditions, and food and water were provided ad libitum. After the experiments, the animals were sacrificed using carbon monoxide. The in vivo study was approved by the Local Ethical Committee of the Animal Experimentation “Comité de Ética de Experimentación Animal de la Xerencia de Xestión Integrada A Coruña (CEEA-XXIAC)” and by the “Consellería do Medio Rural” of Xunta de Galicia (15002/2022/004).

### 2.3. Piglets

Porcine cartilage and synovial membranes were obtained from the joints of 3- to 4-month-old Large White pigs (*Sus scrofa domestica*) with a weight between 30–35 kg. Tissues were flushed with an antibiotic cocktail [penicillin (100 U/mL), streptomycin (100 μg/mL), amphotericin b (4 µg/mL), and gentamicin (4 µg/mL) (Sigma–Aldrich, St. Louis, MO, USA)] and then cultured for 24 h in Dulbecco’s modified Eagle medium (DMEM, Lonza, Basel, Switzerland) with 10% fetal bovine serum (FBS, Gibco, Jenks, OK, USA) and an antibiotic cocktail prior to the addition of mitochondria.

### 2.4. Isolation of Mitochondria

**Figure 1:**
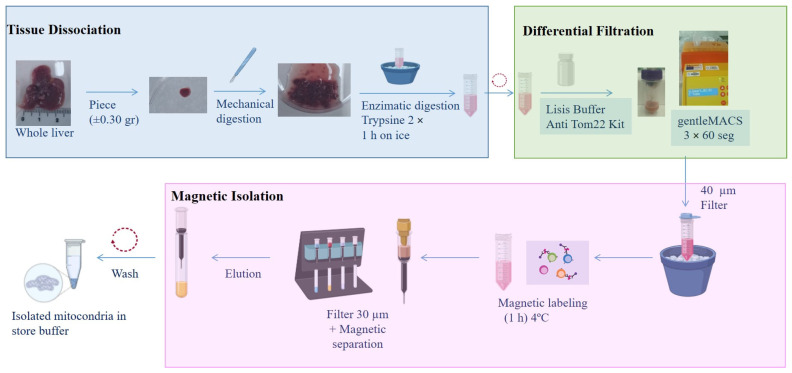
Protocol for isolating mitochondria from liver. A 0.3 g piece of liver was mechanically and enzymatically digested using a gentleMACS, followed by labeling with anti-Tom22 antibody. The isolated mitochondria were preserved in storage buffer.

Mitochondria were isolated from mouse or piglet liver. Briefly, a small piece of tissue was mechanically and enzymatically digested prior to application of the protocol described by Preble and coworkers [40], followed by magnetic purification using anti-TOM22 MicroBeads (Miltenyi Biotec, Bergisch Gladbach, Germany) (Figure 1).

### 2.5. Tissue Dissociation and Differential Filtration

Tissue dissociation and differential filtration were carried out according to the protocol described by Preble [40], with some modifications. Briefly, liver tissue was preserved overnight (O/N) in phosphate-buffer-saline (PBS) supplemented with 10 mM EDTA (PBS + EDTA) (Sigma, Kawasaki, Japan) at 4 °C. The next day, a small piece (±0.35 g) was washed and minced (mechanical digestion). The tissue sample was then homogenized in 2× trypsin (Sigma–Aldrich) for 1 h on ice. The resulting digest was centrifuged at 300× *g* for 10 min, and the pellet was re-suspended in lysis buffer (Anti-TOM22 kit; Miltenyi Biotec) and applied to a dissociation C tube (Gentle MACS C tubes, Miltenyi Biotec). The tube was placed on the gentle MACS^TM^ dissociator (Miltenyi Biotec), and the mitochondrial isolation cycle program was selected (3 × 60 s). The dissociation C tube was moved to an ice bucket, and the resulting homogenate was filtered using a 40 μm mesh filter (Cultek, Madrid, Spain). The elution product was used for the next step.

### 2.6. Isolation of Mitochondria Using Superparamagnetic Microbeads

An Anti-TOM22 MicroBeads kit (Miltenyi Biotec) was used for the magnetic isolation of mitochondria. Microbeads are a colloidal suspension of extremely small (i.e., 50 nm diameter) superparamagnetic particles conjugated to monoclonal anti-TOM22 antibodies. These microbeads were then used for magnetic isolation using a MACS Separator.

The eluate obtained as described in the preceding section was incubated with 50 µL of anti-TOM22 MicroBeads and shaken for 1 h at 4 °C. The suspension was loaded onto a pre-equilibrated MACS Column using the 30 µm filter provided with the kit (Miltenyi Biotec), which was placed in the magnetic field of a MACS Separator (Miltenyi Biotec). After removing the column from the magnetic field, the retained mitochondria was eluted with 5 mL of elution buffer. Following centrifugation at 13,000× *g* for 5 min, the mitochondrial pellet was washed twice prior to resuspension in storage buffer.

### 2.7. Characterization of Isolated Mitochondria

#### Mitochondrial Protein Content

Total mitochondrial protein was obtained using 200 μL of M-PER™ Mammalian Protein Extraction Reagent (Thermo Fisher Scientific, Waltham, MA USA). The protein concentration was determined using a bicinchoninic acid (BCA) assay with a BCA Protein Assay kit (Pierce Biotechnology, Waltham, MA, USA) using a Tecan Infinite M200 spectrophotometer NanoQuant (Tecan, Männedorf, Switzerland). For experiments, 14 or 28 µg of isolated mitochondria was used.

### 2.8. Mitochondrial Purity

#### 2.8.1. Transmission Electron Microscopy (TEM)

Mitochondrial purity and integrity were determined by TEM. Isolated mitochondria were fixed in 2.5% electron microscopy-grade glutaraldehyde in 0.1 M sodium cacodylate (pH 7.4) (Science Services, Bangkok, Thailand) for at least 3 h, then washed 3 times for 5 min each in 0.1 M sodium cacodylate buffer. Afterward, samples were post-fixed in 1% OsO4 in 0.1 M cacodylate for 1 h at room temperature (rt), dehydrated in a graded series of acetone, and embedded in Spurr’ resin (Taab). Semi-thin sections (1 µm thick) were cut using a Reichert–Jung ultramicrotome (Ultracut-E) and stained with toluidine blue and examined under a light microscope. Finally, ultra-thin sections (50–80 nm thick) were stained with uranyl acetate and lead citrate for 5 min and observed and photographed using a JEM1010 transmission electron microscope (Jeol, Tokyo, Japan) at 80 kV.

#### 2.8.2. Western Blotting (WB)

Mitochondrial purity was evaluated by WB. Proteins were extracted from liver homogenate (Lv), the first elution from the magnetic column (Elt), and isolated mitochondria (Mit) using lysis buffer (6 M urea/2% SDS) and sonicated at 4 °C. Protein concentrations were determined using the BCA assay and separated using SDS-PAGE, as previously described [44]. Separated proteins were then transferred to membranes, which were incubated O/N at 4 °C with the following antibodies: mouse anti-ATP synthase α (1:2000; Ab-612516 BD Bioscience, San Jose, CA, USA), rabbit anti-GRP78 BiP (1:1000; Ab-108613 Abcam, Cambridge, UK), anti-α-tubulin (1:3000; Ab-T5168 Sigma–Aldrich) and mouse anti-histone H3 (1B1B2) (1:1000, mAB #14269 cell signaling, Danvers, MA, USA). Anti-rabbit and anti-mouse secondary antibodies (1:1000; GE Healthcare, Chicago, IL, USA) were used. ECL chemiluminescent substrate (Millipore, Burlington, MA, USA) was used to detect antigen–antibody binding with an LAS-3000 luminescent image analyzer (Fujifilm, Tokyo, Japan).

### 2.9. Mitochondrial Viability

#### 2.9.1. MitoTracker Red^®^

Mitochondrial viability was assessed using MitoTracker Red CMXRos (Thermo Fisher Scientific; ref M7512) (MitoTracker Red^®^). Mitochondria were incubated with 250 nM MitoTracker Red^®^ at 37 °C for 30 min, washed three times with saline buffer, and a drop of the suspension was mounted on a slide with Prolong Gold Antifade Reagent. For the negative control, the solution of MitoTracker Red^®^ incubated without mitochondria was used (250 nM MitoTracker Red^®^ was incubated in storage buffer without mitochondria and the same procedure described before was applied). Fluorescence was visualized and photographed with an Olympus BX61 microscope. Red fluorescence intensity was measured using a FACs Calibur flow cytometer. Data analysis was performed using CellQuest software version 5.1.

#### 2.9.2. ATP Production

The metabolic activity of isolated mitochondria was evaluated by measuring the ATP concentration using an ATP assay kit (Perkin Elmer, Waltham, MA, USA) according to the manufacturer’s instructions. Mitochondria were incubated with 0.8 µM rotenone or antimycin A (Sigma) for 2 h before ATP measurement. Luminescence was measured with a Nanoquant Infinite M200Pro (Tecan).

### 2.10. Mitochondrial Labeling

#### 2.10.1. Cy5-Labeled Oligonucleotides

Cy5-labeled oligonucleotides were used to stain mitochondria within liver tissue. Oligo Cy5 at 50 nM was used as described by Lorenz and coworkers [45]. The protocol is described in Figure S1.

#### 2.10.2. Iron Oxide

Isolated mitochondria were incubated with iron oxide (II, III) magnetic nanopowder. A total of 10 mg of N-succinimidyl ester functionalized, 30 nm magnetic iron (II, III) oxide particles (Sigma–Aldrich) were added to 14 µg of isolated mitochondria for 10 min at 0 °C. The suspension was then washed 5 times with storage buffer. To confirm the presence of iron nanoparticles in the samples, TEM was performed, as described in the section Mitochondrial purity.

#### 2.10.3. MitoTracker Red^®^

Isolated mitochondria were labeled with MitoTracker Red^®^. Mitochondria were incubated in the presence of MitoTracker Red^®^ at 37 °C for 30 min. In parallel, a tube containing only storage buffer (without mitochondria) with MitoTracker Red^®^ was prepared as a negative control. The tubes were then washed 3 times by centrifugation at 13,000 rpm for 5 min.

Tissue samples containing MitoTracker Red^®^-labeled mitochondria and negative controls (tissue incubated in the presence of only storage buffer (without mitochondria) with MitoTracker Red^®^) were embedded in Tissue-Tek^®^ optimum cutting temperature (OCT) compound (Sakura^®^ Finetek, Torrance, CA, USA) and snap-frozen. The samples were then cryosectioned and fixed in 4% paraformaldehyde (Sigma-Aldrich) for 10 min at rt, followed by incubation in 0.1% Tween 20 (Sigma–Aldrich) for 5 min. The sections were then incubated for 5 min in a solution of Hoechst 33258 (Sigma–Aldrich) and washed with phosphate buffer (PB). Cover slips were mounted on the microscopy slides using Prolong Gold Antifade Reagent Mountant, and fluorescence was visualized and photographed under a Nikon AR-1 confocal microscope.

### 2.11. Mitochondrial Transplantation

#### 2.11.1. In Vitro Models

Piglet cartilage and synovial membrane were isolated using a 5 mm punch biopsy. Explants were equilibrated in DMEM supplemented with 10% FBS, penicillin (100 U/mL), and streptomycin (100 μg/mL) (Gibco) in a humidified 5% CO_2_ atmosphere at 37 °C. The next day, isolated mitochondria from piglet liver were labelled with MitoTracker Red^®^ or iron nanoparticles and added over the explants. The tissues were then cultured. After incubation, the tissues were fixed in OCT as described in the section Mitochondrial labeling. Tissues incubated with iron nanoparticle-labeled mitochondria were prepared for TEM by fixation in 2.5% electron microscopy-grade glutaraldehyde in 0.1 M sodium cacodylate buffer (pH 7.4) (Science Services) for at least 6 h, then washed 3 times for 5 min each in 0.1 M sodium cacodylate buffer. The remaining steps were carried out as previously described in the section Mitochondrial purity.

#### 2.11.2. In Vivo Models

##### Short-Term Model

C57BL/6JOlaHsd male mice were used. Intra-articular injection into the knee of the animals was performed between the meniscus and the tibial and femoral plateaus using a syringe with a fine needle (Hamilton gauge 29, 10 µL. Sigma) with an optimal angle ensuring entry into the intra-articular space [46]. Given the limited volume of the intra-articular space in the mouse knee, the injected volume was restricted to 10 µL, consistent with volumes reported by other authors [47]. Three experimental conditions were established, and all mice received an intraarticular injection in the left knee:

- Negative control group (Neg); mice were injected with vehicle (only isolation buffer).
- Fluorescence control group (Control); mice were injected with the supernatant obtained from isolation buffer (without mitochondria) incubated with MitoTracker Red^®^ following the same protocol for the Injected mitochondria group.
- Injected mitochondria group (Mito); mice were injected in the joint with isolated mitochondria in isolation buffer labeled with MitoTracker Red^®^.

Forty-eight hours after injection, the animals were euthanized, and the knees and organs were collected, processed, and analyzed for MitoTracker Red^®^ staining. Briefly, knee joints were fixed O/N in 3.7–4% formaldehyde, decalcified by centrifugation in Decal (HistoLab, Brea, CA, USA) for 3 h at 200 rpm, and then subjected to paraffin embedding. Serial sections (4 μm) were cut, deparaffinized in xylene, and then subjected to a graded series of alcohol washes. The organs were fixed in OCT and processed as described above in the section Mitochondrial labeling. Fluorescence was visualized and photographed under an Olympus microscope.

##### Long-Term Model

Healthy (without any surgery or OA) male C57BL/6JOlaHsd mice received a single intraarticular injection of mitochondria obtained from C57BL/6JOlaHsd or NZB/OlaHsd mice to evaluate auto- and allogenic responses. Three experimental conditions were established and all mice in each group received an injection in the left knee. Mice were injected with isolated mitochondria obtained from C57BL/6JOlaHsd (Mitoc57) or NZB/OlaHsd (MitoNZB) mouse strains, and control mice were injected with vehicle (Control). After 7 weeks, the animals were sacrificed, and knee joints and organs were obtained and analyzed as described in the previous section Mitochondrial labeling. Body weight and joint width were measured weekly. Cartilage, synovial damage and inflammation were assessed according to the Osteoarthritis Research Society International (OARSI) score [48] and Krenn classification [49,50], respectively.

### 2.12. Statistical Analyses

Data are presented as mean ± standard error of mean (SEM) from at least three independent experiments, with a minimum of three observations unless stated otherwise. Statistical analyses were performed using GraphPad Prism software, version 8.0.1. The unpaired nonparametric Mann–Whitney test was used to evaluate differences between experimental conditions. Differences with *p* values ≤ 0.05 were considered statistically significant.

## 3. Results

### 3.1. Characterization of Isolated Mitochondria

**Figure 2:**
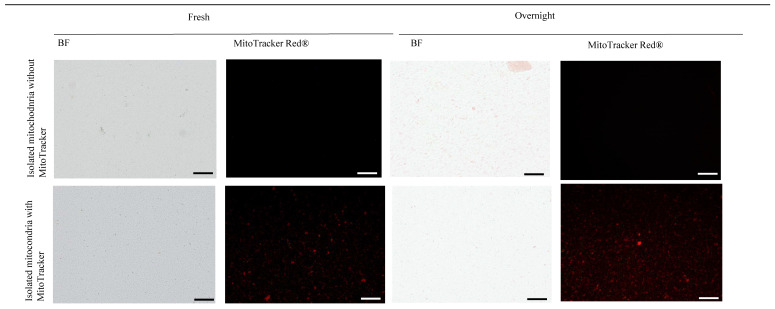
Liver preserved at 4 °C in PBS+EDTA overnight. Mouse livers were obtained and used fresh or preserved in PBS+EDTA (10 mM) overnight (O/N) before mitochondrial isolation using a previously described protocol. Isolated mitochondria were stained with MitoTracker Red^®^, and the organelles were analyzed using bright-field (BF) and fluorescence microscopy. Scale bar: 100 μm.

To determine if the mitochondria remained viable when the tissues from which they were isolated are preserved beforehand, liver specimens were kept O/N at 4 °C in PBS+EDTA. Mitochondria were subsequently isolated, and their viability was assessed using MitoTracker Red^®^. The fluorescence intensity did not decrease over time in comparison with analysis of mitochondria obtained from fresh samples (Figure 2). Based on this result, the following experiments were performed with liver samples preserved O/N.

**Figure 3:**
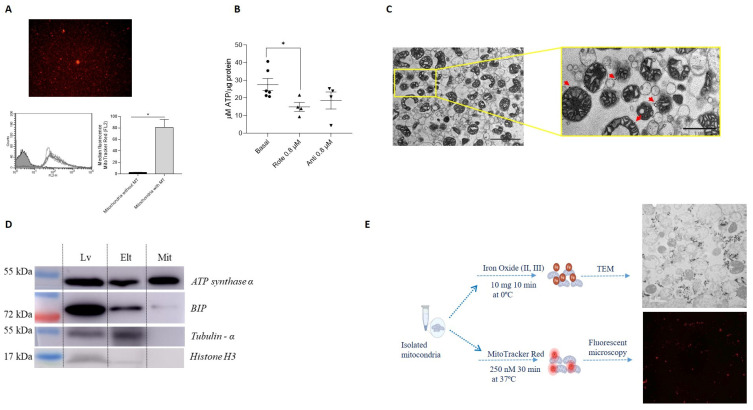
Purity and viability of isolated mitochondria. (**A**) Membrane potential (ψm) of isolated mitochondria labeled with MitoTracker Red^®^. Representative fluorescence microscopy image is shown (**upper panel**). FACS analysis was conducted to compare mitochondria without and with MitoTracker Red^®^ (MT) staining, and the results are presented as graphs showing the median fluorescence (**lower panels**) (*n* = 4). (**B**) ATP production (nmol/µg mitochondrial protein) was determined using an ATP assay of isolated mitochondria under basal conditions or incubated with 0.8 µM rotenone (Rot) or antimycin A (Ant) for 2 h (*n* = 4). (**C**) Transmission electron microscopy (TEM) image of isolated mitochondria. Representative image of mitochondria from mouse liver. Mitochondrial morphology was investigated using TEM, and images were acquired at magnifications of 9000× (**left panel**) and 25,000× (**right panel**). Black bars indicate 2.5 µm (**left panel**) and 1 µm (**right panel**). Black dots at the outer membrane represent anti-TOM22 magnetic beads (red arrows). (**D**) Western blotting of protein extracts probed with antibodies specific for organelle/cell compartment–specific marker proteins (ATPsintase α), the endoplasmic reticulum (GRP78-BIP), cytosol (α-Tubulin) and nucleus (Histone H3). Lv: liver homogenate, Elt: first elution through the magnetic column, Mit: isolated mitochondria. * *p* ≤ 0.05. (**E**) Iron nanoparticle and MitoTracker Red^®^ labeling of isolated mitochondria. Mitochondria were isolated from mouse liver and then labeled with iron nanoparticles (**upper panel**) or MitoTracker Red^®^ (**lower panel**).

Mitochondrial viability was further evaluated by analyzing membrane potential using flow cytometry and measuring ATP production, confirming that the isolated mitochondria were functional. The measures evaluating mitochondrial membrane potential (Figure 3A) and ATP production, and its reduction by inhibitors of the electron transport chain (Figure 3B), indicated that the mitochondria obtained were functional.

Mitochondrial purity was determined using transmission electron microscopy (TEM) and western blot (WB) (Figure 3C,D). TEM images of isolated mitochondria are shown in Figure 3C. Analysis of the purity of the mitochondrial fraction by WB showed that the isolated mitochondria had higher levels of ATP synthase α and lower levels of ER marker (GRP78-BiP is one of the most abundant chaperones and is a key component of the endoplasmic reticulum (ER) [51,52] and was used as an ER marker) in comparison with protein fractions obtained from the liver or the magnetic column eluate. In contrast, the cytoplasmic (tubulin-α) and nucleus (histone H3) components were not detected in the mitochondrial fraction (Figure 3D).

### 3.2. Mitochondrial Labeling

Isolated mitochondria were labeled using three different methodologies to determine the best option for subsequent analysis after transplantation in in vitro and in vivo models. In liver samples stained with Cy5-labeled oligonucleotides, the presence of Cy5 did not affect the tissue morphology and enabled labeling of the mitochondrial network within the tissue. However, the fluorescence intensity decreased upon subsequent isolation of the mitochondria (Figure S1). Organelles were also labeled with iron nanoparticles or MitoTracker Red^®^, showed positive signals in both cases (Figure 3E). As a result, both stains were used for labeling isolated mitochondria in the following experiments.

### 3.3. Mitochondrial Administration in In Vitro Models

**Figure 4:**
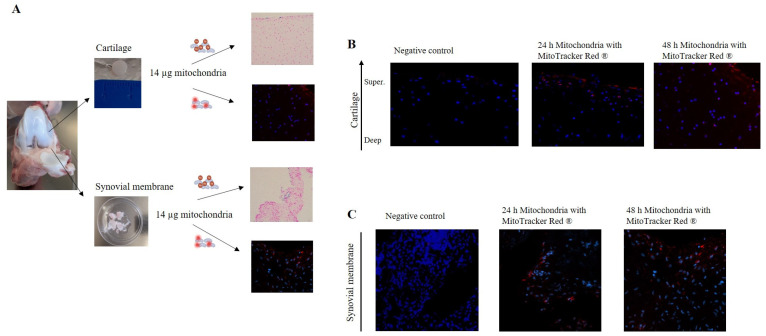
Mitochondria have the capacity to break through joint tissue. (**A**) Piglet model. Cartilage (**upper panels**) and synovial membrane (**lower panels**) were obtained and incubated in the presence of 14 µg of mitochondria, obtained from piglet liver, labeled with iron nanoparticles or MitoTracker Red^®^. (**B**) Cartilage tissue was incubated in the presence of isolated mitochondria labeled with MitoTracker Red^®^ for 24 and 48 h and then analyzed using confocal microscopy. (**C**) Synovial membrane was incubated according to the same procedure described above. Negative control = tissue incubated with storage buffer (without mitochondria) with MitoTracker Red^®^. Representative images 20×.

To evaluate the capacity of mitochondria to integrate into joint tissues, cartilage and synovial membrane explants were co-incubated with isolated mitochondria (14 µg) labeled with iron nanoparticles or MitoTracker Red^®^. Labeled mitochondria obtained from piglet liver were added to piglet cartilage and synovial membrane explants and incubated for 24 or 48 h (Figure 4A). When tissues were incubated in the presence of only storage buffer (without mitochondria) with MitoTracker Red^®^ (Negative control), no red fluorescence was detected (Figure 4B,C). However, when tissues were co-cultured in the presence of isolated mitochondria, a fluorescence signal was observed and its intensity was maintained in both tissues for 24 and 48 h (Figure 4B,C). Notably, analysis of the cartilage images showed the presence of mitochondria in the superficial and intermediate layers (Figure 4B).

**Figure 5:**
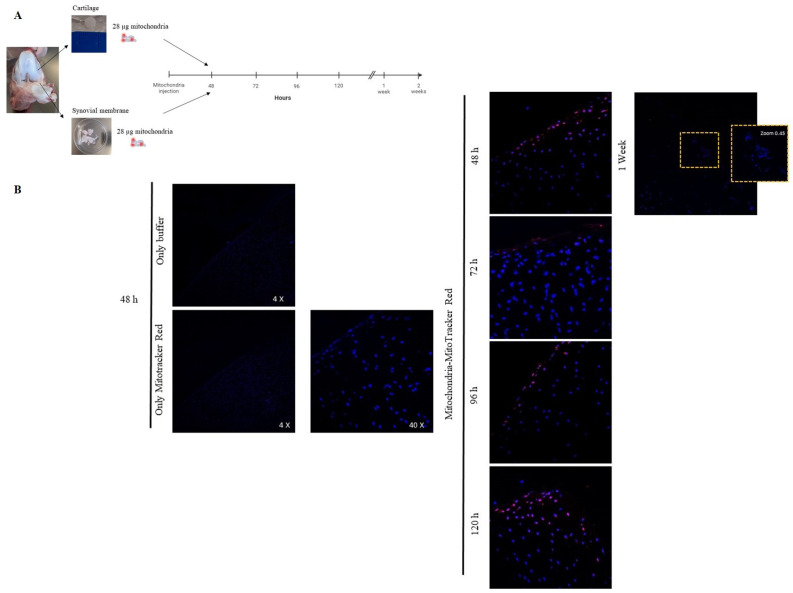
Confirmation that mitochondria have the capacity to break through joint tissue. (**A**) Piglet model. Joint tissues were obtained and incubated in the presence of 28 µg of mitochondria (obtained from piglet liver) labeled with MitoTracker Red^®^ and incubated for 48, 76, 96, 120 h and 1 and 2 weeks. (**B**) Cartilage tissue was incubated in the presence of only buffer and only MitoTracker Red^®^ using as technique controls, then isolated mitochondria labeled with MitoTracker Red^®^ were incubated during the time described and the tissue was analyzed using confocal microscopy.

**Figure 6:**
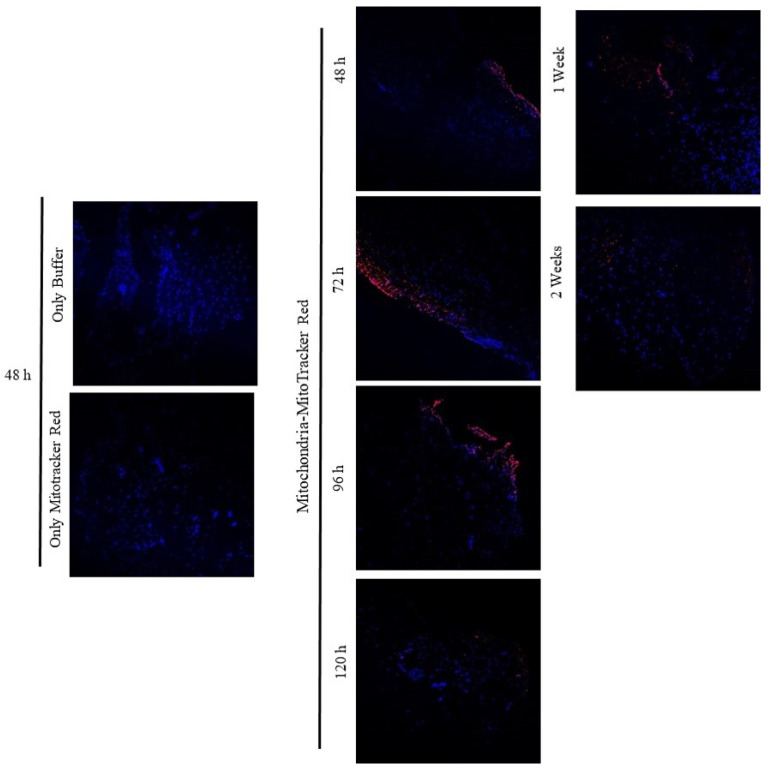
Confirmation that mitochondria have the capacity to break through the synovial membrane. Synovial membrane was incubated in the presence of only buffer and only MitoTracker Red^®^ using as technique controls, then isolated mitochondria labeled with MitoTracker Red^®^ were incubated during the time described and the tissue was analyzed using confocal microscopy. Representative images 40×.

Based on these data, we replicated the experiment, increasing the concentration of isolated mitochondria injected to 28 µg and the incubation time (Figure 5A). Analysis of the cartilage images indicated that viable mitochondria were detected from 48 h to 1 week after incubation (Figure 5B). Mitochondria labeled with MitoTracker Red^®^ in the synovial membrane were detected for up to 2 weeks (Figure 6).

**Figure 7:**
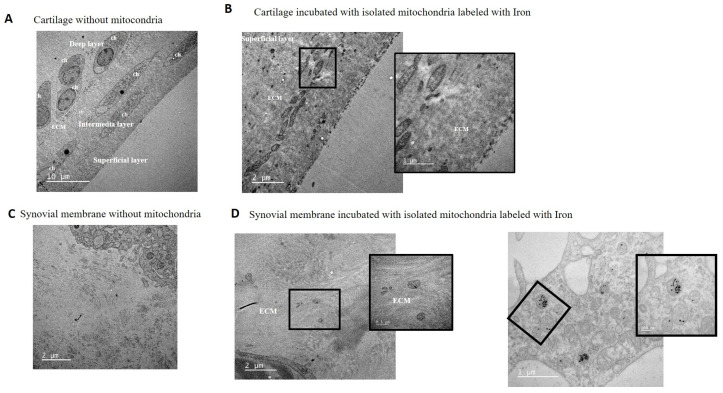
Mitochondria were detected in cartilage and synovial membrane using TEM. (**A**) Representative TEM images of cartilage explants without added mitochondria showed no mitochondria in the ECM. The panel shows the chondrocyte morphology in the different cartilage layers. Images were acquired at 5000×, and the white bar indicates 10 µm. (**B**) Representative TEM images of cartilage explants with added mitochondria labeled with iron nanoparticles for 48 h. Left panel shows the presence of mitochondria in the superficial layer embedded in the ECM. Images were acquired at 15,000× and 40,000×, respectively, and the white bars indicate 2 and 1 µm, respectively. (**C**) Synovial membrane without added mitochondria showed synoviocytes and ECM morphology investigated by TEM; image was acquired at 15,000×, and white bars indicate 2 µm. (**D**) Representative image of synovial membrane incubated in the presence of mitochondria labeled with iron nanoparticles showing mitochondria in the ECM and inside some synoviocytes. **Left panel** shows the presence of mitochondria embedded in the ECM and magnification of a small area; these images were acquired at 15,000× and 50,000×, respectively, and the white bars indicate 2 µm and 0.5 µm, respectively. **Right panel** shows the presence of mitochondria labeled with iron nanoparticles inside the synoviocytes and the magnification of a small area; these images were acquired at 25,000× and 60,000×, respectively, and the white bars indicate 2 µm and 200 nm, respectively. ECM = extracellular matrix, ch = chondrocyte, s = synoviocyte.

To confirm the capacity of mitochondria to penetrate joint tissues, samples from the in vitro model were analyzed using TEM with iron nanoparticle labeling. TEM analysis of cartilage without mitochondria did not show any mitochondria in the extracellular matrix (ECM) (Figure 7A). TEM images of cartilage incubated for 48 h with mitochondria showed the presence of isolated mitochondria in the superficial layer of the tissue, embedded in the ECM of the cartilage (Figure 7B).

The capacity of isolated mitochondria to incorporate into the cells and ECM of the synovial membrane was also analyzed by microscopy. The morphology of the tissue and major cells, synoviocytes, were evaluated before mitochondrial injection and confirmed the absence of extracellular mitochondria in the ECM (Figure 7C). Analysis of the synovium after 48 h of incubation showed the presence of isolated mitochondria and iron nanoparticles in the ECM and within some synoviocytes (Figure 7D).

### 3.4. Mitochondrial Injection in the Short-Term In Vivo Model

**Figure 8:**
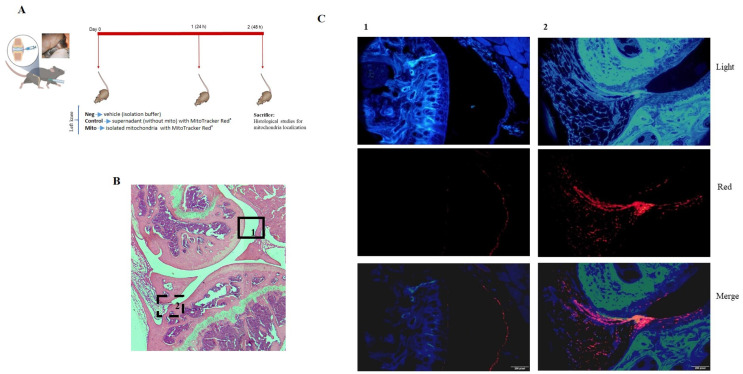
In vivo model. (**A**) Mitochondria obtained from mouse liver were labeled with MitoTracker Red^®^ and injected into the mouse’s left knee; after 48 h, the animal was sacrificed and the left joint was obtained and processed. (**B**) Representative image of complete left knee stained with hematoxylin and eosin (H&E). (**C**) Complementary images from areas 1 (black box in (**B**)) and 2 (dash box in panel (**B**)) analyzed under fluorescence microscopy. Red fluorescence from isolated mitochondria (red) was detected in the superficial layer of the cartilage and in the synovial membrane. Light: image obtained without fluorescence. Red: image obtained whit red fluoresce.

**Figure 9:**
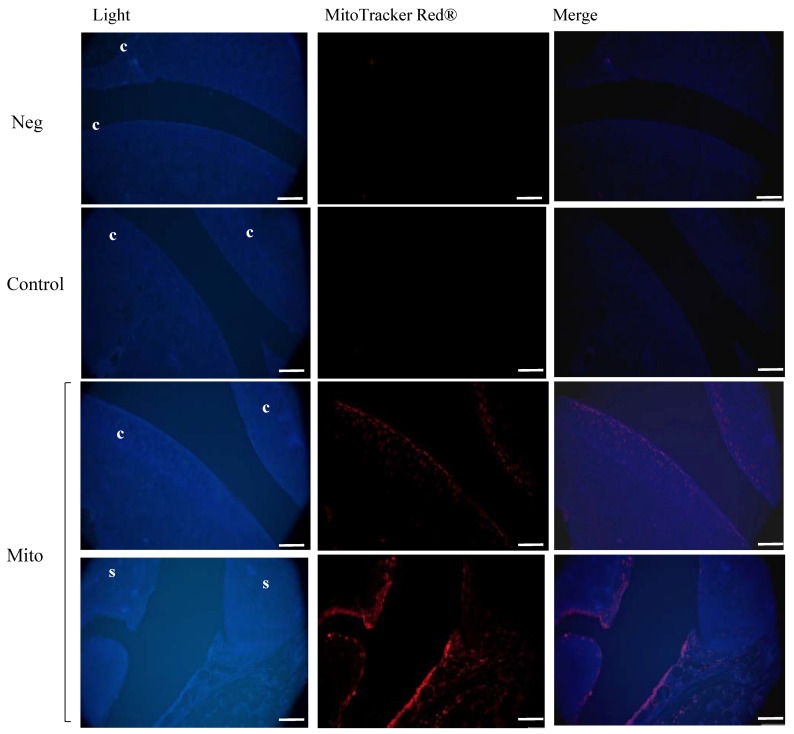
In vivo model analysis of red fluorescence showed the presence of red mitochondria in the superficial layer of the cartilage and in the synovial membrane. Neg = vehicle (only isolation buffer) inject into the joint, Control = supernatant obtained from isolation buffer (without mitochondria) incubated with MitoTracker Red^®^, Mito = isolated mitochondria in isolation buffer labeled with MitoTracker Red^®^, c = cartilage, s = synovial membrane. Scale bar, 400 µm.

A short-term in vivo model was developed using C57BL/6JOlaHsd mice in which viable mitochondria labeled with MitoTracker Red^®^ were injected into the left knee and the animals were euthanized 48 h later (Figure 8A). To determine the location of the injected viable mitochondria in the joint, red fluorescence was analyzed in the articular tissues. As shown in Figure 8B,C and Figure 9, a noticeable red signal was detected in the superficial layer of the cartilage and in the synovial membrane compared to the negative control (vehicle-injected joint) or MitoTracker control (MitoTracker Red^®^-injected joint), in which no red fluorescence was detected. No infiltration of mitochondria was observed by monitoring red fluorescence in the internal organs (Figure S2). Therefore, we concluded that the injected mitochondria had not been displaced into the bloodstream in our short-term in vitro model.

### 3.5. Mitochondrial Injection in the Long-Term In Vivo Model

**Figure 10:**
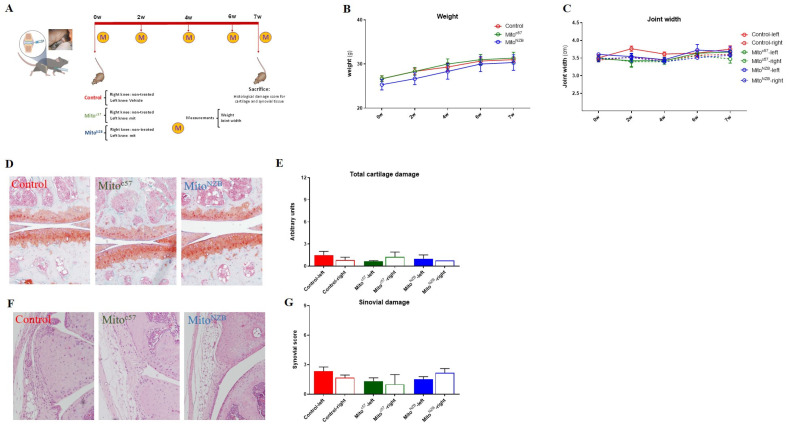
In vivo model. (**A**) Schematic illustration of the long-term in vivo model. Mitochondria obtained from C57BL/6JOlaHsd (MitoC57) and NZB/OlaHsd (MitoNZB) mice were injected in the left knee of healthy C57BL/6JOlaHsd mice (without OA damage). Control mice received no injection (Control). After 7 weeks, the mice were sacrificed. M = measurements each week. (**B**) Weight during the 7 weeks. (**C**) Joint width. (**D**,**E**) Representative staining of cartilage with Safranin O. OARSI score to evaluate cartilage damage. (**F**,**G**) Representative staining of synovial membrane with H&E. Krenn score to evaluate synovial membrane inflammation/damage. Full boxes correspond to the left knee, and the empty boxes represent the right joint. *n* = 3 animals per group.

To evaluate the long-term effects of intra-articular injection of mitochondria as well as allogeneic responses in the joint, C57BL/6JOlaHsd (autologous; Mitoc57) or NZB/OlaHsd (allogeneic; MitoNZB) mitochondria were injected into C57BL/6JOlaHsd mice (Figure 10A). To assess model safety, body weight and joint width were measured weekly for 7 weeks, revealing that administration of C57BL/6JOlaHsd or NZB/OlaHsd mitochondria did not adversely affect these parameters relative to non-injected C57BL/6JOlaHsd (Control) mice (Figure 10B,C). Histological analysis of the internal organs showed no detectable signs of damage (Figure S3). Evaluation of cartilage and synovial damage and inflammation signals showed that injection of isolated mitochondria from the same or different strain induced neither pathological changes in the cartilage (Figure 10D,E) nor synovial inflammation (Figure 10F,G). We did not observe differences in the response when comparing the data from the injected knee (left) with the contralateral knee (right). These data indicated that the intra-articular injection of isolated mitochondria of autologous or allogeneic origin was safe in mice and did not damage the joint tissues and did not induce an inflammatory response in the joint.

## 4. Discussion

This work sets the stage for the study of a novel therapeutic based on the intra-articular injection of isolated mitochondria. Our results demonstrated the feasibility and safety of this approach, providing a foundation for future investigations into the therapeutic potential of mitochondrial transplantation in joint tissues within an osteoarthritis (OA) model.

The first objective was to develop a robust protocol for isolating high-purity, viable mitochondria suitable for intra-articular transplantation. Numerous procedures have been described in the literature that involve various parameters affecting the quality and viability of isolated mitochondria [53,54]. We adopted and modified the procedure described by Preble and coworkers [40], combining mechanical and enzymatic digestion with magnetic purification using anti-TOM22 microbeads to increasing the purity of the mitochondria isolated. However, the method described here slightly increases the time required for the isolation of mitochondria but the obtained mitochondria are viable and pure, which facilitates their use in mitochondrial injection, particularly in both in vivo and in vitro models.

This protocol allowed us to obtain isolated mitochondria with high viability after preserving the tissue overnight, thereby facilitating its implementation in clinical practice. This method allowed us to achieve mitochondria with high purity as evidenced by the lack of contamination with endoplasmic reticulum and cytosolic markers in the mitochondrial fractions. Transmission electron microscopy (TEM) and Western blot (WB) analyses confirmed the structural integrity and purity of the isolated mitochondria.

Several options are available for labeling isolated mitochondria. Oligonucleotides labeled with Cy5 or Cy3 have been utilized as mitochondrial markers in different cell lines but have not been previously used in tissues [45,46,47,48,49,50,51,52,53,54,55]. In our study, we demonstrated that Cy5-labeled oligonucleotides could effectively label mitochondria within the tissue; however, upon isolation from the tissue, the mitochondria exhibited low red fluorescence signals. Iron nanoparticles also offer a viable option for labeling mitochondria [56]. By labeling isolated mitochondria with 30 nm iron oxide particles, we obtained critical information regarding the distribution of transplanted mitochondria in joint tissue.

The functionality of the isolated mitochondria was rigorously tested through several assays. MitoTracker Red^®^ staining demonstrated that the mitochondria retained their membrane potential over time, indicating preserved viability. Additionally, ATP production assays confirmed the metabolic activity of the mitochondria, further supporting their functionality. These findings are crucial as they validate the use of these mitochondria for subsequent transplantation experiments.

Several authors have reported the successful transfer of exogenous mitochondria into cells or tissues, using MitoTracker dyes to trace these mitochondria within the recipient cells [57,58]. In this study, labeling mitochondria with MitoTracker was utilized to confirm the internalization of exogenous mitochondria in various models [57,58,59]. In vitro experiments with piglet cartilage and synovial membrane explants provided valuable insights into the integration capabilities of the isolated mitochondria. When co-cultured with mitochondria labeled with MitoTracker Red^®^ or iron nanoparticles, the explants exhibited significant fluorescence signals, indicating successful incorporation of mitochondria into the superficial and intermediate layers of cartilage as well as the synovial membrane. Several authors detected iron-labeled mitochondria in porcine hearts after injection, but this did not confirm their viability [17,18,19,20,21,22,23,24]. In contrast, our study showed viable mitochondria labeled with MitoTracker Red a week post-injection, suggesting mitochondrial viability, although further studies will be need to confirm this. TEM analysis further corroborated these findings, showing mitochondria embedded in the extracellular matrix (ECM) and within some synoviocytes.

In the short-term in vivo model, intra-articular injection of MitoTracker Red^®^-labeled mitochondria resulted in detectable red fluorescence in the superficial layer of the cartilage and the synovial membrane 48 h post-injection. Importantly, no red fluorescence was observed in the internal organs, suggesting that the mitochondria remained localized within the joint. This localization is critical for minimizing potential systemic effects and maximizing therapeutic efficacy.

Previous studies have shown a lack of immune response following the injection of autogenetic and allogeneic mitochondria [60,61,62]. To assess the long-term safety and potential allogeneic responses, we injected mitochondria isolated from both autologous (C57BL/6JOlaHsd) and allogeneic (NZB/OlaHsd) sources into C57BL/6JOlaHsd mice. Over a seven-week period, no adverse effects on body weight or joint width were observed, and there were no signs of damage in internal organs. Our findings are consistent with recent studies that have shown that the administration of allogeneic mitochondria from healthy animals into skeletal muscle of dystrophin-deficient mdx mice did not produce adverse effects and demonstrated that systemic mitochondrial transplantation therapy alleviates the progression of destructive processes in the muscle tissue of these mice [22]. Histological analyses revealed no pathological changes in cartilage or synovial tissue, indicating that the intra-articular injection of mitochondria, whether autologous or allogeneic, was safe and did not induce inflammatory responses in the joint.

## 5. Conclusions

This study established a foundational protocol for functional mitochondrial isolation and demonstrated that isolated mitochondria can penetrate the various tissues that constitute the joint, as demonstrated by our in vitro experiments. In vivo, isolated mitochondria injected into the joint penetrated the superficial layer of cartilage and the synovial membrane, remaining in the joint for at least 48 h post-injection. Additionally, this study indicates that mitochondrial injection is safe, does not damage the cartilage or synovium, and does not cause systemic toxicity. These findings support the potential of mitochondrial transplantation as a therapeutic strategy for OA as well as other diseases with mitochondrial dysfunction. However, further studies are necessary to elucidate the long-term effects of mitochondrial transplantation, optimal dosing strategies, and the underlying mechanisms by which transplanted mitochondria may confer therapeutic benefits.

## Data Availability

Data sharing is not applicable to this article.

## Supplementary Materials

The following supporting information can be downloaded at: https://www.mdpi.com/article/10.3390/cells14030151/s1. Figure S1: Staining of liver using Cy5-labeled oligonucleotides. Figure S2: Spleen and liver from in vivo mouse model of mitochondrial injection. Figure S3: Liver, kidney, and spleen from in vivo mouse model of mitochondrial injection.
