# Supplementary material for: In Vivo and In Vitro Evaluation of the Feasibility and Safety Profiles of Intraarticular Transplantation of Mitochondria for Future Use as a Therapy for Osteoarthritis
Source: Cells. 2025 Jan 21;14(3):151. doi: 10.3390/cells14030151 (PMC11817340; doi:10.3390/cells14030151)
Supplement: Supplementary file 1 [file cells-14-00151-s001.zip › cells-3343949-supplementary.pdf]

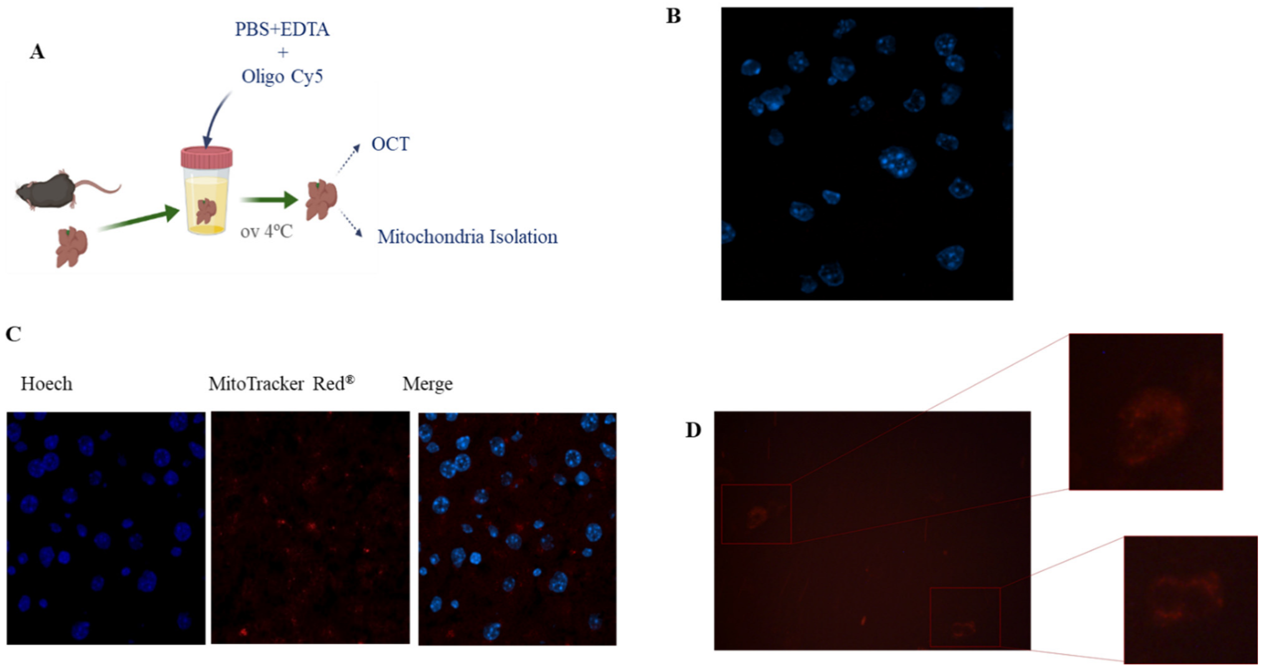

Figure S1 Staining of liver using Cy5-labeled oligonucleotides. Oligo Cy5 (5'-Cy5- CCA CTC CTT TCC AGA AAA CTA TTG CAA ATG ACG GATCCG C-3') at 50 nM was used. Liver was incubated O/N at 4°C in PBS+EDTA solution with the oligo. After incubation, a piece of the tissue was embedded in OCT compound (Sakura® Finetek) and snap-frozen. Samples were cryosectioned and stored at −80°C until use. Another fragment was used for mitochondria isolation following the protocol described above. **A.** Schematic illustration of the procedure for staining liver tissue with oligo Cy5. **B.** Representative image corresponding to liver incubated O/N in PBS+EDTA and staining with Hoechst; this image corresponds to the negative condition, showing that the incubation did not affect tissue morphology. **C.** Representative image corresponding to liver incubated overnight in PBS+EDTA with oligo Cy5; the slide stained with Hoechst demonstrated the use of Cy5 to label the mitochondrial network inside the tissue. **D.** Isolation of mitochondria following the protocol described here from liver incubated O/N in presence of oligo Cy5, reflecting weak fluorescence. O/N = overnight.

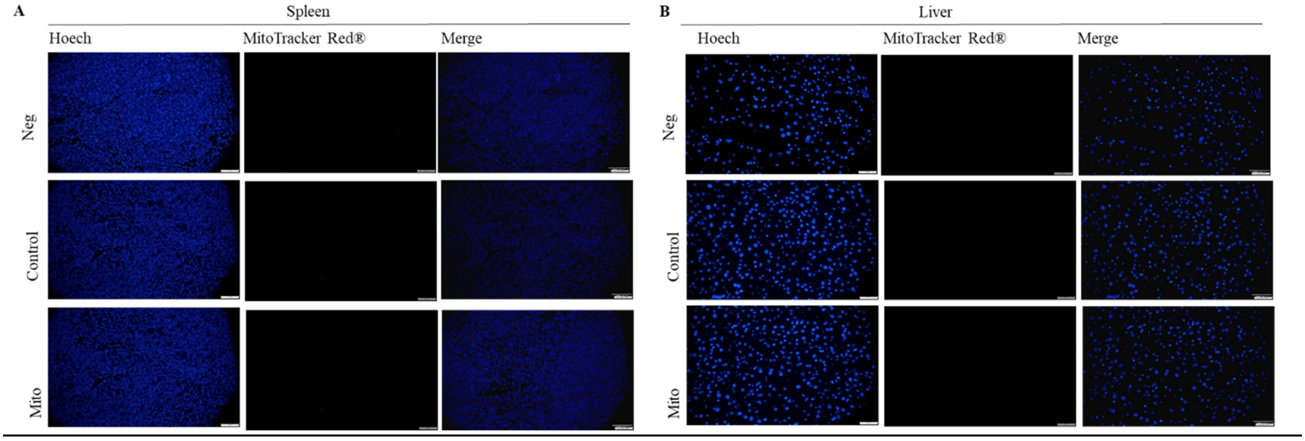

Figure S2. Spleen and liver from *in vivo* mouse model of mitochondrial injection. Representative images corresponding to spleen and liver from mice injected with mitochondria labeled with MitoTracker Red®. The analysis of red images showed negative signals; these data indicated that the mitochondria did not have the capacity to translocate to distant organs from the knee, and from the same analysis, we concluded that mitochondrial injection did not generate toxicity. Neg = vehicle (only isolation buffer) inject into the joint, Control = supernadant obtained from isolation buffer (without mitochondria) incubated with MitoTracker Red®, Mito = isolated mitochondria in isolation buffer labeled with MitoTracker Red®

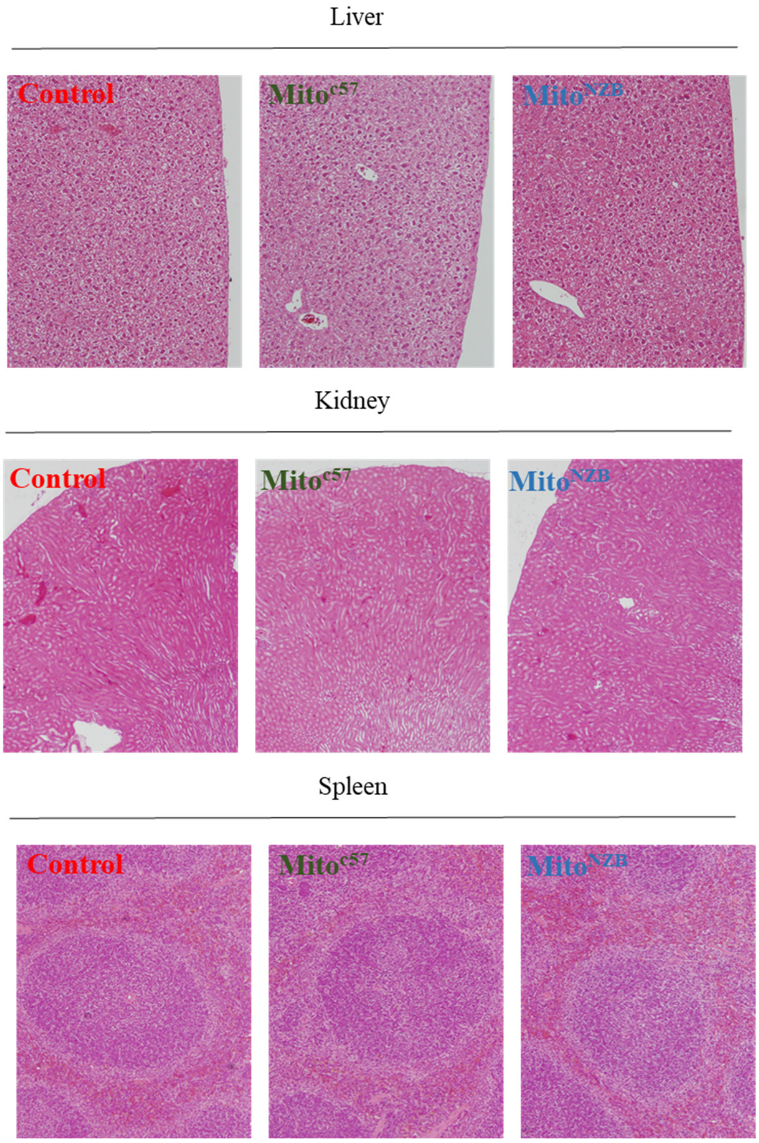

Figure S3. Liver, kidney, and spleen from *in vivo* mouse model of mitochondrial injection. Representative images corresponding to liver, kidney, and spleen from C57BL/6JOlaHsd mice injected with mitochondria obtained from C57BL/6JOlaHsd (Mito^{C57}) and NZB/OlaHsd (Mito^{NZB}) mice and without injection (Control). The image analyses did not show any tissue alterations in the organs analyzed.
